# c-Jun N-terminal Kinase Supports Autophagy in Testicular Ischemia but Triggers Apoptosis in Ischemia-Reperfusion Injury

**DOI:** 10.3390/ijms251910446

**Published:** 2024-09-27

**Authors:** Sarah R. Alotaibi, Waleed M. Renno, May Al-Maghrebi

**Affiliations:** 1 Department of Biochemistry, College of Medicine, Kuwait University, Safat 13110, Kuwait; sarah.alotaibi@grad.ku.edu.kw; 2 Department of Anatomy, College of Medicine, Kuwait University, Safat 13110, Kuwait; waleed.renno@ku.edu.kw

**Keywords:** autophagy, oxidative stress, JNK, molecular pathway, spermatogenesis, testis, ischemia reperfusion injury

## Abstract

Oxidative stress triggered by testicular torsion and detorsion in young males could negatively impact future fertility. Using a rat animal model for testicular IRI (tIRI), we aim to study the induction of autophagy (ATG) during testicular ischemia and tIRI and the role of oxidative-stress-induced c-Jun N-terminal Kinase (JNK) as a cytoprotective mechanism. Sixty male Sprague-Dawley rats were divided into five groups: sham, ischemia only, ischemia+SP600125 (a JNK inhibitor), tIRI only, and tIRI+SP600125. The tIRI rats underwent an ischemic injury for 1 h followed by 4 h of reperfusion, while ischemic rats were subjected to 1 h of ischemia only without reperfusion. Testicular-ischemia-induced Beclin 1 and LC3B expression was associated with decreased p62/SQSTM1 expression, increased ATP and alkaline phosphatase (AP) activity, and slightly impaired spermatogenesis. SP600125 treatment improved p62 expression and reduced the levels of Beclin 1 and LC3B but did not affect ATP or AP levels. The tIRI-induced apoptosis lowered the expression of the three ATG proteins and AP activity, activated caspase 3, and caused spermatogenic arrest. SP600125-inhibited JNK during tIRI restored sham levels to all investigated parameters. This study emphasizes the regulatory role of JNK in balancing autophagy and apoptosis during testicular oxidative injuries.

## 1. Introduction

Autophagy is an established regulatory mechanism of spermatogenesis, germ cell differentiation, and testosterone biosynthesis [1]. Thus, it is vital to the male reproductive system. Consequently, dysregulated autophagy is associated with impaired spermatogenesis and male infertility. Our research aims to understand the role of autophagy in testicular oxidative injuries. This could hold the potential to provide solutions to several male reproductive pathologies resulting in low sperm quality and infertility associated with dysregulated autophagy [2].

Testicular torsion is a urologic emergency that affects young males who have a subfertility rate of 36–39% after detorsion [3]. Although it is a curative approach, the detorsion-prompted re-flow of nutrients and oxygen to the ischemic testis generates an environment of oxidative stress (OS) within the seminiferous tubules (STs). To study the underlying cause of male subfertility induced by testicular torsion and detorsion (TTD), we used a rat model of unilateral testicular ischemia reperfusion injury (tIRI). Previous experimental investigations identified arrested spermatogenesis, oxidative DNA damage, ER stress, and germ cell apoptosis as key consequences of tIRI [4,5]. Similar testicular OS-induced dysregulations were also reported in association with the activation of autophagy [6]. Furthermore, somatic and germ cells were found to express protein components of the autophagy pathway, and several autophagy-deficient organisms produce a variety of reproductive abnormalities or sometimes cause infertility [7]. While germ cell apoptosis was a hallmark in our tIRI animal model, the role of autophagy ought to be investigated as an equally important regulatory pathway of cell survival and death.

Autophagy is usually activated during stressful events to degrade and recycle damaged nutrients to maintain cellular homeostasis and promote organ survival [8]. It is a catabolic pathway responsible for sequestrating undesirable macromolecules and targeting them for lysosomal degradation. Upon autophagy induction, Beclin 1-dependent autophagosome nucleation and maturation provide an interaction platform for lipid kinases and other autophagy proteins. Beclin 1 is also a trafficking protein that transfers the mature lapidated LC3B to the phagophore’s outer membrane. Increased tethering of LC3B will recruit several proteins to trigger autophagosome expansion and completion. Thus, Beclin 1 and LC3B are bona fide biomarkers for autophagosome numbers and, thus, autophagic flux. Ubiquitinated proteins are sequestered from the cytoplasm to autophagosomes by binding to the scaffold protein p62/sequestosome 1 (p62/SQSTM1). The ubiquitinated p62 acts as an autophagy receptor that binds tightly to LC3B at the autophagosome’s inner membrane to sequester undesired protein aggregates destined for autophagic degradation. The formation of autolysosomes leads to the targeted degradation of p62 and its cargo. Thus, the accumulation of p62 is known to be a general marker of reduced autophagic flux. Within autolysosomes, acid phosphatases and hydrolases will degrade the cytoplasmic components destined for autophagic clearance. However, increased lysosomal acid activity or irreparable lysosomal membrane damage could trigger cellular death.

Autophagy is a double-edged sword that acts as a pro-survival mechanism during stressful conditions, but it can also induce autophagic cell death. Uncontrolled autophagy prompts the cell to use it as a cellular death signal if conditions of starvation caused by ischemia or OS are prolonged [9]. Though they have different execution mechanisms as pathways of regulated cell death, apoptosis and autophagy share some modulating proteins that suggest the presence of crosstalk between them [10]. The two pathways can either be activated simultaneously or operate sequentially during cellular stress. Both pathways are vital to maintaining testicular homeostasis by regulating germ cell development via the removal of defective germ cells to produce normal mature gametes [11]. The c-Jun N-terminal kinase (JNK) is a stress-activated protein kinase that has been reported to regulate tIRI-induced germ cell apoptosis, and it has been linked to the regulation of autophagy [12,13]. Therefore, JNK could be another common signaling component between apoptosis and autophagy. The JNK/autophagy axis has been studied in several organs but not in the testis.

OS-induced apoptosis is triggered by the sudden oxygen influx during tIRI. Here, we propose that stress-induced autophagy could occur during testicular ischemia due to the lack of oxygen and nutrients as a pro-survival pathway. Thus, this study aims to monitor the expression of autophagy biomarkers and common apoptosis–autophagy components during testicular ischemia and tIRI injuries using a rat model. The role of JNK in autophagy regulation will be investigated using its specific inhibitor SP600125.

## 2. Results

### 2.1. JNK Phosphorylation during Testicular Ischemia and IR Injuries

**Figure 1:**
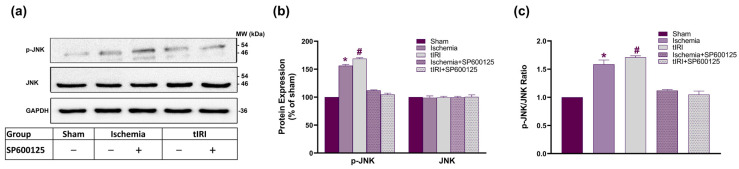
JNK phosphorylation is associated with testicular ischemia and tIRI. (**a**) Representative Western blots (WB) of JNK and p-JNK (T183 and Y185) in ipsilateral testes of experimental groups: sham, ischemia, tIRI, ischemia+SP600125, and tIRI+SP600125. SP600125 is injected intraperitoneally 30 min prior to ischemia and tIRI at 15 mg/Kg. (**b**) WB band intensity of JNK and p-JNK normalized to GAPDH. (**c**) Ratio of p-JNK to JNK expression. Data are presented as mean ± SD (*n* = 6/group), *p*-value < 0.05. * Ischemia compared to sham and # tIRI compared to sham.

The effect of testicular ischemia and tIR injuries on JNK activation was investigated using WB analysis (Figure 1). Compared to the sham, the expression of p-JNK increased by 156% and 169% in the ischemia- and tIRI-subjected testis, respectively. However, there was no measurable change in the level of total JNK expression. The calculated p-JNK to JNK ratio was 1.59 and 1.71 for ischemia and tIR injuries, respectively. Treatment with SP600125 reverted p-JNK expression and the phosphorylation ratio to sham levels in the two testicular injuries. Contralateral testes showed no significant changes in JNK phosphorylation (*p*-value > 0.05). This indicates the activation of JNK during testicular ischemia and tIRI and its inhibition by SP600125.

### 2.2. Effect of JNK on the Expression of Autophagy Markers Beclin 1, LC3B, and p62

**Figure 2:**
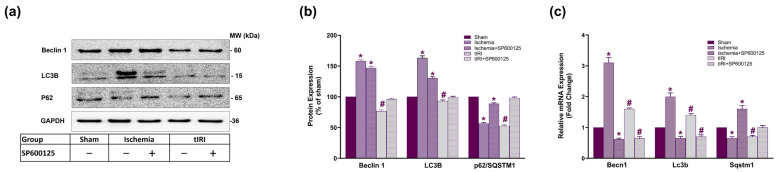
JNK modulates the gene expression of autophagy markers: Beclin 1, LC3B, and p62. (**a**) Representative Western blots and (**b**) bar graphs of Beclin 1, LC3B, and p62/SQSTM1 protein expression in all 5 experimental groups. (**c**) The relative mRNA expression of autophagy markers Becn1, Lc3b, and Sqstm1 was calculated using the 2^−ΔΔCt^ method. The fold change in gene expression in ischemia-, tIRI-, and SP600125-treated groups was calculated relative to the sham group. * Ischemia compared to sham and # tIRI compared to sham.

Autophagy flux was detected by examining the protein expression of its markers, Beclin 1, LC3B, and p62, which represent initiation, elongation, and execution, respectively, using WB analysis (Figure 2a,b). During testicular ischemia, the protein expression of Beclin 1 and LC3B was induced by 158% and 163%, respectively, while p62 expression was reduced by 56.7% compared to the sham (*p* < 0.0001). SP600125-mediated JNK inhibition decreased the expression of Beclin 1 and LC3B to 147% and 130%, respectively, but it increased p62 to 88.7% compared to the sham (*p* < 0.0001). During tIRI, Beclin 1, LC3B, and p62 protein levels were reduced to 76.7%, 93%, and 52.7%, respectively, compared to the sham (*p* < 0.0001). Following SP600125 treatment, the protein expression of the three autophagy proteins was at sham levels. This indicates that ischemia-induced JNK is partly responsible for regulating the protein expression of the three autophagy markers. However, other pathways are likely involved in maintaining autophagy during testicular ischemia to preserve testicular function.

At the transcriptional level (Figure 2c), the relative mRNA expression of *Becn1* and *Lc3b* was upregulated during testicular ischemic injury by 3.1- and 2-fold, respectively, while *Sqstm1* (p62) mRNA expression was downregulated by 1.54-fold compared to the sham (*p* < 0.0001). JNK inhibition reversed this expression pattern to 1.54-fold downregulation for *Becn1* and *Lc3b* and 1.6-fold upregulation for *Sqstm1* mRNA (*p* < 0.0001). During tIRI, the transcription rate of the *Becn1* and *Lc3b* genes was increased by 1.6- and 1.4-fold; however, *Sqstm1* was decreased by 1.43-fold compared to the sham (*p* < 0.0001). SP600125 treatment during tIRI normalized *Sqstm1* mRNA expression but downregulated that of *Becn1* and *Lc3b* by 1.43- and 1.54-fold, respectively (*p* < 0.0001). This suggests that JNK is capable of directly regulating the mRNA expression of autophagy genes via activation of its downstream transcription factors.

**Figure 3:**
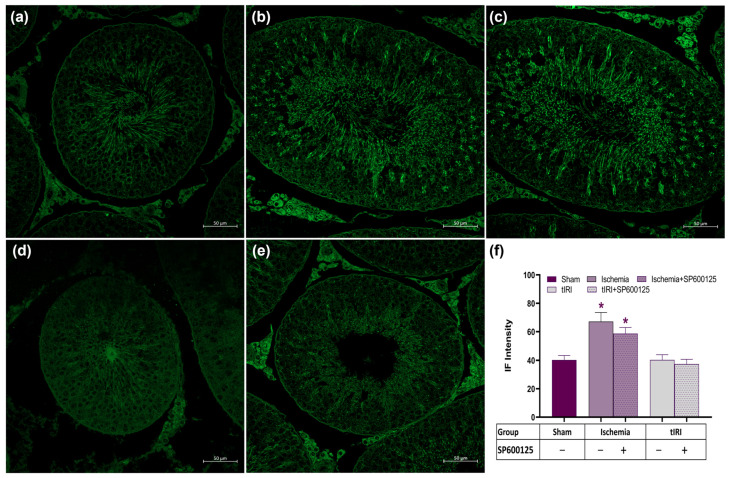
JNK modulates LC3B expression. LC3B was detected in seminiferous tubules using fluorescence confocal microscopy. Representative immunofluorescence (IF)-stained testicular tissue sections for (**a**) sham, (**b**) ischemia, (**c**) ischemia+SP600125, (**d**) tIRI, and (**e**) tIRI+SP600125 groups. (**f**) Quantification of the average IF intensity and statistical analysis. Images were taken at 40× magnification with a scale bar of 50 μm. Data are presented as mean ± SD (*n* = 6/group), *p*-value < 0.05. * Ischemia compared to sham.

Autophagy flux was also monitored qualitatively and quantitatively by detecting LC3B immunofluorescence (IF) signals as they associated with the autophagosome membrane (Figure 3). The LC3B IF signal was increased by 1.67- and 1.45-fold during testicular ischemia and following JNK inhibition with SP600125 compared to the sham, respectively (*p* < 0.0001). However, baseline IF signals of LC3B were observed and calculated during tIRI and after SP600125 treatment. This shows the formation of LC3B structures during autophagy flux in the testis under ischemic conditions but not during tIRI. LC3B IF structures were localized to haploid germ cells like spermatozoa and spermatids (elongated and round). This could suggest that during ischemia-induced nutrient deficiency, the number of meiotic germ cells is held constant by the decreased nurturing capacity of Sertoli cells, which limits the number of spermatogonia entering meiosis.

### 2.3. Effect of JNK on AP Activity and ATP Levels

**Figure 4:**
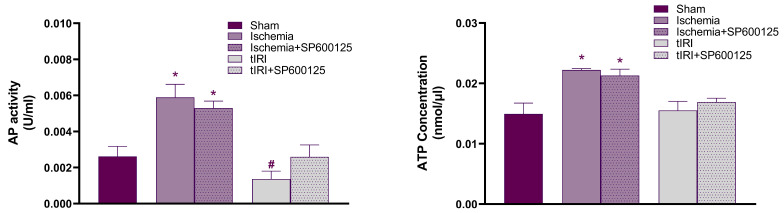
JNK modulates lysosomal alkaline phosphatase (AP) activity but not ATP levels. Biochemical colorimetric assay kits measured AP activity and ATP concentration in the 5 experimental groups. The data were analyzed using one-way analysis of variance (ANOVA) followed by the Holm–Sidak multiple comparisons test and presented as mean values ± SD (*n* = 6/group). * Ischemia compared to sham and # tIRI compared to sham.

During ischemia, the activity of the lysosomal AP enzyme was increased by 2.25- and 4.30-fold in comparison to sham and tIRI levels, respectively (*p* < 0.0001) (Figure 4). SP600125-mediated JNK inhibition during ischemia reduced AP activity; however, it remained higher than the sham and tIRI groups by 2- and 3.86-fold, respectively (*p* < 0.0001). During tIRI, AP activity decreased by 0.52-fold compared to sham levels (*p* < 0.0001), which was normalized by SP600125 inhibition of JNK (*p* = 0.0961). Contralateral testes showed no significant changes in AP activity (*p*-value > 0.05). Although AP activity is negatively impacted by JNK activation in testicular ischemia and tIRI, its activity is likely sustained by other autophagy-supporting pathways during ischemia.

ATP concentration was elevated during ischemia by 1.5-fold compared to sham and tIRI levels (*p* = 0.0007 and *p* = 0.0011, respectively) (Figure 4). Sham and tIRI testes had comparable ATP concentrations (*p* = 0.7221). Ischemia-induced ATP levels were not affected by SP600125 treatment and showed a 1.43-fold increase over sham and tIRI groups (*p* = 0.0017 and *p* = 0.0027, respectively). Sham-like ATP levels in tIRI were also not affected by JNK inhibition (*p* = 0.4210). Contralateral testes showed no significant changes in ATP concentration (*p*-value > 0.05). The data rule out the regulatory effect of JNK on ATP cellular levels during testicular oxidative injuries.

### 2.4. Effect of JNK on Caspase 3 Activity

**Figure 5:**
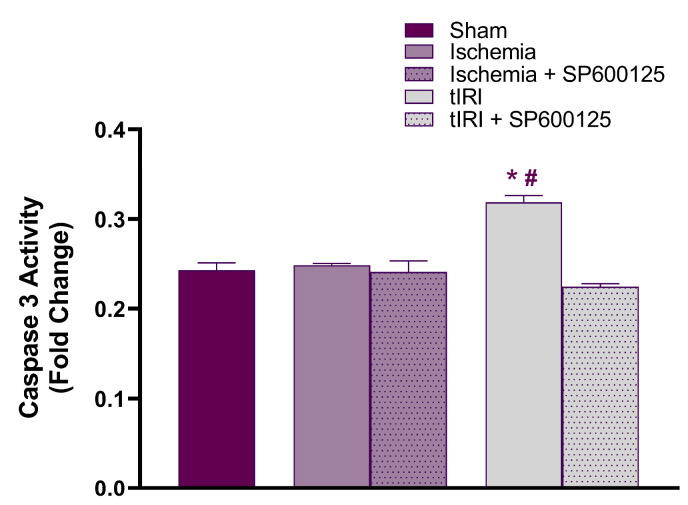
JNK modulates caspase 3 activity. Caspase 3 activity was measured using a colorimetric assay, and the fold change in ischemia-, tIRI-, and SP600125-treated groups was calibrated to sham activity. Data are presented as mean ± SD (*n* = 6/group), *p*-value < 0.05. * tIRI compared to ischemia and # tIRI compared to sham.

In testicular ischemic injury, caspase 3 activity was at sham levels (*p* = 0.6085) and not affected by SP600125 treatment (*p* = 0.7433) (Figure 5). However, a 1.3-fold increase in caspase 3 activity was calculated during tIRI compared to the sham and ischemia injury only (*p* < 0.0001). This increase was normalized through SP600125 treatment (*p* = 0.0517). Contralateral testes showed no significant changes in caspase 3 activity (*p*-value > 0.05). This indicates that apoptosis is inhibited by ischemia-induced autophagy, possibly by blocking the activation of caspase 3 or its initiator caspases.

### 2.5. Effect of JNK on Spermatogenesis

**Figure 6:**
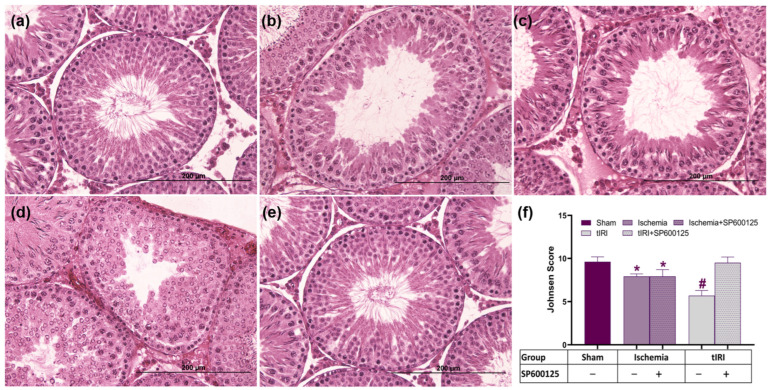
JNK modulates spermatogenesis activity. H&E-stained testicular tissue sections for (**a**) sham, (**b**) ischemia, (**c**) ischemia+SP600125, (**d**) tIRI, and (**e**) tIRI+SP600125 groups. (**f**) Spermatogenesis analysis using the Johnsen score. Images were taken at 40× magnification with a scale bar of 200 μm. Data are presented as mean ± SD (*n* = 6/group), *p*-value < 0.05. * Ischemia compared to sham and # tIRI compared to sham.

Histological examination demonstrated a slight spermatogenic impairment in ischemia, which was arrested in tIRI compared to the sham (7.92 ± 0.29, 5.69 ± 0.60, and 9.62 ± 0.57, respectively; *p* < 0.0001). SP600125 treatment had no effect on spermatogenesis in ischemia, but it normalized it in tIRI (7.91 ± 0.79 and 9.50 ± 0.66, respectively) (Figure 6). A Johnsen score of 5–6 indicates the loss of spermatozoa and the presence of few spermatids, a score of 7–8 indicates the presence of few spermatozoa and late spermatids, and a score of 9–10 indicates the presence of mature sperms. The tIRI-subjected testis showed more spermatogenic damage than those enduring an ischemic injury only. This implies the harmful additive consequences of tIRI-induced oxidative stress and germ cell apoptosis. Together with the IF findings, it seems that the autophagic haploid germ cells are more vulnerable to undergoing apoptosis, as shown by their absence in the STs. Contralateral testes showed no significant changes in the Johnsen score (*p*-value > 0.05). The data also demonstrate the dual role of JNK in balancing cellular survival and death to preserve the quality of germ cells and support testicular function. It acts as a pro-survival pathway during ischemia-induced nutrient starvation and it promotes autophagy, but it switches to a cell-death-signaling pathway in tIRI-induced OS due to excessive ROS generation.

## 3. Discussion

The current study provides evidence for the dual action of JNK signaling to balance autophagy and apoptosis during an acute testicular ischemic event and at the early stages of tIRI. The obtained data suggest that while the ischemic testis can support its survival via autophagic recycling of macromolecules, it is overridden by the overwhelming influx of oxygen and nutrients during tIRI. The ischemia autophagic survival mode is switched to apoptotic cell death mode as a protective pathway against tIRI-induced OS under the regulation of JNK. This molecular switch provides an efficient testicular response mechanism to harmful stress conditions that could impede the sensitive process of spermatogenesis.

ROS-induced OS is a well-established culprit in male infertility disorders. Growing evidence correlates ROS production with the incidence of autophagy, where oxidative intermediates are upstream modulators of autophagy [14]. Thus, ROS can inhibit autophagy by either oxidizing autophagy redox sensors or inactivating upstream autophagy modulators. JNK activation is one of the first lines of cellular stress responses to ROS accumulation. In response to OS, the mammalian ATG9 protein is essential for JNK activation by complexing with TRAF6 [15]. Dissociation of the ATG9/TRAF6 complex negatively regulates the ROS-induced JNK and blocks its downstream-activated ATG, thus inhibiting autophagy. Its vital role in modulating cellular survival and death is extensively investigated and demonstrated by its ability to participate in apoptotic and autophagic events in several tissues subjected to stressful conditions. This dual role is propagated through its downstream transcription factors, like c-jun, ATF, Nrf2, and FoxO, that enhance or repress the expression of apoptosis and autophagy-related genes [16]. Studies have also shown that JNK regulation of autophagy components extends to the post-transcriptional level. Previously, we showed that JNK activation induced germ cell apoptosis during tIRI and was partly responsible for the excessive accumulation of ROS, DNA damage, and impaired spermatogenesis [13]. However, the dual-signaling role of JNK prompted us to investigate whether it could also have cross-action with the autophagy pathway during tIRI and acute testicular ischemia. Autophagic events and their modulation have been reported in several organ models of IRI of the brain, heart, and liver, with suggested participation of JNK signaling [17,18,19]. In cadmium-exposed spermatocytes, JNK inhibition with SP600125 attenuated autophagic flux and induced autophagosome-dependent apoptosis [20]. Similarly, chemically induced reproductive injury in male rats resulted in impaired spermatogenesis associated with increased autophagy and apoptosis via the IRE2/JNK signaling pathway [21]. Sertoli cells exposed to 4-Nonylphenol suffered from reproductive dysfunction due to the simultaneous induction of apoptosis, necrosis, and autophagy in the ROS-mediated activation of JNK [22]. In both testicular ischemia and tIRI, JNK phosphorylation and activation was observed, which was effectively inhibited by SP600125 treatment.

The regulatory role of JNK in autophagy induction was monitored by examining the expression of its main biomarkers: Beclin 1, LC3B, and p62. Beclin 1 upregulation is essential for autophagy, but, physiologically, it is suppressed by its interaction with the anti-apoptosis Bcl2 [23]. The role of JNK in modulating apoptosis and autophagy was first described in cells’ responses to nutrient availability [24]. JNK-phosphorylated Bcl2 at multiple sites was undoubtedly the key regulator of autophagy and apoptosis. Acute starvation conditions caused JNK to phosphorylate the ER pool of Bcl2 and dissociate from Beclin 1 to induce autophagy. However, persistent starvation caused JNK to phosphorylate the mitochondrial pool of Bcl2 at other sites, forcing the breakage of its strong interaction with the pro-apoptosis Bax molecule and thus triggering apoptosis. Subsequently, an in vitro hypoxia model also showed that autophagy but not cell death was induced via the action of the mitochondrial autophagy receptor BNIP3, which disrupts Bcl2/Beclin 1 interaction and releases Beclin 1 [25]. BNIP3 was later established as a phosphorylation substrate of JNK, through which it can induce ER stress, mitophagy, and apoptosis [26]. Trophoblast cells with a BNIP3 knockdown were shown to have impaired mitophagy due to the suppression of Beclin 1 and LC3-II and the overexpression of p62, which indirectly suggests the blocking of JNK signaling [27]. A recent report demonstrated that JNK-induced mitophagy was mediated via its phosphorylation of BNIP3, which enhanced its interaction with LC3 and resulted in an increased LC3-II/I ratio and the formation of LC3 puncta [28]. These observations were reversed by JNK inhibition using SP600125 and siRNA knockdown. Furthermore, hypoxia-induced p62 degradation was inhibited by autophagy inhibitors and attenuated LC3 expression [29]. Overall, autophagy is considered to be a cytoprotective mechanism employed by ischemic tissues to support their survival. In this study, SP600125 treatment normalized the expression of p62 during ischemia, while Beclin 1 and LC3B overexpression was reduced but remained higher than sham levels. It could be suggested that Beclin-1-dependent autophagy could persist in the absence of JNK. During nutrient loss, it was shown that AMPK phosphorylates Beclin 1 at Thr388 in the BARA domain to induce autophagy [30]. This suggests that JNK promotes cell survival during ischemia via autophagic recycling of macromolecules to maintain testicular homeostasis. However, it could be speculated that prolonged or chronic ischemia could deplete cellular contents, and the now-protective autophagy pathway could switch to a death pathway [31]. Under ROS-induced OS and JNK activation, Beclin 1 is cleaved by caspase 3, leading to autophagy inhibition and the induction of apoptosis [32]. As part of the Beclin 1 interactome, LC3B is also influenced by JNK signaling during ROS-induced OS. JNK inhibition alleviated cardiac IRI-induced p62 degradation and overexpression of Beclin 1 and LC3-II and triggered apoptosis [12]. Beclin 1 deletion in these cells suppressed autophagy induction. These reports agree with our current findings. In tIRI-subjected testes, Beclin 1, LC3B, and p62 expression levels were all reduced due to the JNK-induced germ cell apoptosis and increased caspase 3 cleavage of Beclin 1. In addition, inhibition of JNK during tIRI led to the normalization of Beclin 1, LC3B, and p62 expression, suggesting JNK’s regulatory influence over autophagy. 

Transcriptionally, JNK was shown to regulate the gene expression of autophagic genes via its downstream transcription factors, FoxO [33]. Like JNK, FoxO factors are involved in apoptotic and autophagic cell deaths. Suppression of FoxO1 expression resulted in decreased autophagosome formation due to the inhibition of p62 degradation and LC3B accumulation. Its cytoplasmic overexpression caused autophagy induction and autophagic cell death [34]. This shows that FoxO is indispensable for autophagy induction. Other studies have revealed that the induction of autophagic genes like *Lc3* and *Bnip* in IR injuries and starvation was the result of FoxO3 activity and not FoxO1 [35,36]. JNK also phosphorylates c-jun, which translocate to the nucleus to activate Beclin 1 gene expression, thus promoting autophagy [17]. Additionally, the autophagy receptor p62 complexes with keap1, a negative regulator of Nrf2, and sequesters it in autophagosomes for degradation, leading to Nrf2 activation. During renal OS, SP600125 treatment inactivated JNK, suppressed keap1, and activated Nrf2, leading to enhanced p62 mRNA expression [37]. Data from this study revealed the downregulation of *Sqstm1/p62* mRNA and the upregulation of *Becn1* and *Lc3b* mRNAs in both testicular ischemia and tIRI. Inhibition of JNK by SP600125 treatment reversed the mRNA levels of the three genes. Any differences in the ATG mRNA and protein expression could be attributed to regulation at the post-transcriptional and post-translational levels of autophagic proteins. 

Notably, Beclin1, LC3B, and p62 were reported to facilitate apoptosis besides autophagy. This emphasizes the presence of a functional relationship between the two pathways, especially under pathophysiological stimuli. The Bcl2/Beclin 1 complex is a well-established and key molecular rheostat regulating autophagy and apoptosis in mammalian systems [38]. The interaction of LC3B with the extrinsic apoptosis factor Fas regulated cigarette-smoke-induced apoptosis after detecting low apoptosis levels in the lungs of LC3B^−/−^ mice [39]. Caspase 8 is an initiator caspase that activates the executioner caspase 3 through cleavage to execute the final stages of apoptosis. During autophagy, caspase 8 activity is selectively abolished through lysosomal degradation to prevent apoptosis [40]. The pro-caspase 8 can be complexed with ATG16L1 and drawn to the autophagosome surface by p62 [41]. Through self-polymerization, p62 sequesters ubiquitinated caspase 8 into aggresomal structures, thus preventing ionizing-radiation-induced apoptosis [42]. LC3 and p62 aggresomes were also shown to promote apoptosis via the mediation of caspase 8 activation [43,44]. This explains the lack of caspase 3 activation during testicular ischemia, which coincided with autophagy induction and possible caspase 8 degradation. However, tIRI-induced JNK activation and germ cell apoptosis were associated with heightened caspase 3 activity, which was normalized through SP600125 treatment. Thus, under ROS-induced OS, testicular function (i.e., spermatogenesis and steroidogenesis) depends on the crosstalk between these two pathways and how their components work together or against each other to regulate testicular cell survival or death [45]. Based on the findings from our earlier studies and the present study, we propose that JNK activation during autophagy and apoptosis acts as a molecular rheostat. JNK can sense the tipping point between germ cell survival and death and is able to switch between them. In ischemia-induced autophagy, JNK activation was accompanied by the accumulation of LC3B structures in meiotic germ cells. These are the same cells that underwent apoptosis during tIRI and were absent from the histological organization of seminiferous tubules. Ischemia-induced Beclin-1 expression is suggested to increase caspase 3 activity, implying that enhanced autophagy could induce apoptosis [31]. Here, increased caspase 3 activity was demonstrated in tIRI but not during testicular ischemia. Although no direct evidence was provided in this study, the lack of a significant increase in the levels of lipid peroxidation after 60 min of testicular ischemia without reperfusion confirms the lack of excessive ROS generation [46]. Thus, it could be suggested that JNK activity induced through ischemia supports autophagy in the absence of testicular ROS and OS, but sustained JNK activity during tIRI-induced OS triggered apoptosis [5,13]. Together, it is plausible that despite its complexity, extreme autophagy may promote apoptosis, especially in the vulnerable microenvironment of the testis.

Spermatogenesis consumes many nutrients to support its dynamics, making it vulnerable to pathophysiological stimuli, especially those resulting in starvation and OS. During tIRI-induced OS, ATP production by glycolysis is cellularly favored over mitochondrial (m) ATP production due to excessive mROS production, which lowers mATP production via OXPHOS. In response to low ATP levels, the metabolic activator kinase AMPK switches on fatty acid oxidation, glycolysis, and autophagy pathways to restore cellular ATP reserves and switches off ATP-consuming pathways [47]. AMPK maintains mitochondrial integrity during cardiac IRI and negatively modulates JNK activity and its downstream NF-kB signaling [48]. Glucose starvation for 2 h triggered AMPK to generate energy by phosphorylating autophagic proteins to promote autophagy [49]. This agrees with our findings, as ATP levels were increased during ischemia but not affected during tIRI. Additionally, the inhibition of JNK did not affect ATP levels, as both are controlled by the upstream AMPK [48].

Physiologically, the male reproductive system utilizes autophagy to maintain germ cell homeostasis, promote normal development, and contribute to spermatogenesis [50]. Thus, basal autophagy is considered a cytoprotective mechanism for spermatogenesis. This is attributed to the dynamic nature of autophagy, which enables cells to adapt to stressful environments and simultaneously preserves reproductive function. Several studies have confirmed the involvement of autophagy at each stage of spermatogenesis physiologically and in pathophysiological conditions [51]. The effects of disrupted autophagic flux have been reported in several male reproductive disorders, like oligospermia, azoospermia, asthenozoospermia, teratozoospermia, and globozoospermia. [52]. Patients with a history of cryptorchidism were reported to have increased autophagy rates in their sperm cells in contrast to the lack of autophagic vacuoles in their normozoospermic counterparts [53]. Because autophagy is an active process in human spermatozoa, its effect on sperm quality and motility is possible, which could impact its fertilization capacity [54]. Varicocele-induced male infertility is also characterized by heightened levels of autophagy in the testis and mature sperms compared to control subjects [55]. A rat model of long-term testicular torsion and detorsion showed a significant increase in autophagic indices in somatic and germ cells [56]. This study showed that spermatogenesis was slightly impaired during ischemia and arrested during tIRI, which was associated with the induction of autophagy and apoptosis, respectively. SP600125 treatment suggests that JNK triggered Beclin-1-induced autophagy and caspase-3-dependent apoptosis during ischemia and tIRI, respectively.

The current results broadened our knowledge of the molecular survival mechanisms used by the male reproductive system during ischemia and OS. JNK is identified as a modulator kinase of the molecular switch between autophagy and apoptosis in male germ cells by regulating common proteins. Thus, it could contribute to developing new pharmaceutical therapies for male infertility. The pro-survival role of autophagy during testicular ischemia can be translated into clinical research for the precise targeting of autophagy proteins as potential diagnostic biomarkers in the future.

## 4. Materials and Methods

### 4.1. Animals and Experimental Groups

Adult male Sprague-Dawley (SD) rats (Charles River, Waltham, MA, USA) were housed in chambers with controlled temperature and light and dark cycles lasting 12 h each. Water and standard rat food were provided without restriction. Animal welfare and surgical procedures were conducted following the Health Sciences Center’s policies regarding the ethical use of laboratory animals and animal care (approval code MG-23-07). Rats were randomly divided into five groups (*n* = 12/group, 8 weeks old, 250–300 g): (1) sham, (2) ischemia only, (3) ischemia+SP600125, (4) tIRI only, and (5) tIRI+SP600125.

### 4.2. Rat Model of Unilateral tIRI

We used an established unilateral tIRI rat model, as described previously [13]. Briefly, rats were anesthetized and intraperitoneally injected with a mixture of Ketamine (50 mg/kg; 0143-9508-10; Hikma Pharmaceuticals, Amman, Jordan) and Xylazine (2 mg/kg; XYLO-100, Jaapharm Canada Inc., Vaughan, ON, Canada). An ilioinguinal incision was made, and the left testis was exposed. A straight bulldog clamp with 700 g pressure was used to occlude the spermatic artery of the left ipsilateral testis for 60 min. In the ischemia-only group, the testes were harvested 60 min after testicular artery occlusion and prior to clamp removal. In the tIRI group, the clamp was removed after 60 min of ischemia, initiating testicular reperfusion. Rats were sacrificed 4 h after reperfusion, and both testes were harvested. Sham controls underwent the same surgical procedure but without vascular occlusion. Testes used for biochemical assays, gene expression analysis, and Western blots were cut, flash-frozen in liquid nitrogen, and stored at −80 °C. For histological analyses, testes were immediately placed in Bouin’s fixative for further processing and the preparation of paraffin blocks. For all experiments, the left ipsilateral testis was used for inducing ischemia and tIR injuries, while the right contralateral testis served as the internal control and left untouched in its scrotal sac.

### 4.3. Treatment Protocol

Rats were treated with the JNK inhibitor SP125600 (15 mg/kg intraperitoneally (i.p.); S1460; Selleck Chemicals, Houston, TX, USA) or 10% DMSO as a vehicle. The SP600125 dosage and mode of administration were chosen based on their use in previously published studies using rat IRI models with established statistically significant inhibition of JNK phosphorylation [13,57,58,59]. In the ischemia-only group, the drug was injected 30 min prior to the ischemic insult. In the tIRI group, the drug was injected 30 min after ischemia initiation. The drug injection time was based on the reported maximum serum availability and inhibitory effect of SP600125, which is 30 min after i.p. injection [60].

### 4.4. Hematoxylin and Eosin (H&E) Staining and Johnsen Score

Rehydrated testicular paraffin sections (5 μm) were stained with hematoxylin followed by eosin (H&E) prior to mounting. Using light microscopy (Carl Zeiss AG, Oberkochen, Germany), images of 30 STs per rat group were captured at 40× magnification. Testicular damage was measured using the Johnsen scoring system (1–10) based on the progressive disappearance of somatic and germ cells within the seminiferous tubules [61]. A score of 10 indicates full spermatogenesis, and 1 indicates the loss of Sertoli and germ cells.

### 4.5. Fluorescence Confocal Microscopy Detection

The citrate buffer antigen retrieval protocol was used for the LC3B immunofluorescence (IF) staining. Testicular sections (5 μm) were dewaxed and rehydrated before immersing in a citrate buffer (0.01 M, pH 6.0). The slides were then microwaved for 4 min and cooled to room temperature. PBS-washed slides were blocked with 5% goat serum (G9023, Sigma/Merck, Darmstadt, Germany) and 1 drop of triton X-100 (T9284, Sigma/Merck, Darmstadt, Germany) for 1 h to prevent non-specific sites. Slides were then incubated overnight with MAP LC3b primary antibody (1:100 dilution; sc-271625; Santa Cruz Biotechnology, Dallas, TX, USA) at 4 °C in a moist, dark chamber. PBS-washed slides were incubated with the Alexa Fluro 488 secondary antibody (1:200 dilution; A11029; Thermo Fisher Scientific, Waltham, MA, USA) for 1 h at room temperature in a moist, dark chamber. Finally, the PBS-washed slides were DAPI-mounted, covered, and sealed. IF-stained slides were visualized with the Zeiss LSM 980 confocal microscope Airyscan 2 (Carl Zeiss AG, Oberkochen, Germany), and the fluorescence intensity of 20 STs per group was measured using the ZEN System 3.3 (Carl Zeiss AG, Oberkochen, Germany).

### 4.6. Western Blot Analysis

Testicular tissues were homogenized in lysis buffer on ice. Total protein extracts (350 μg/lane) were separated using 10–12% SDS-PAGE. The resolved proteins were immobilized onto 0.2 μm PVDF membranes (#1620177; Bio-Rad, Hercules, CA, USA), and blots were blocked in 5% non-fat dried milk. Next, the membranes were incubated overnight with the corresponding primary antibody at 4 °C. PBS-washed blots were incubated with a 1:2000 diluted secondary antibody (133499/133599; Jackson ImmunoResearch Labs, West Grove, PA, USA) for 1 h at room temperature. Protein bands were visualized using the ECL detection reagents (RPN2106; Thermo Fisher Scientific, Waltham, MA, USA), and their intensities were quantified using the Chemidoc imaging system (Bio-Rad; Hercules, CA, USA). The primary antibodies for JNK (sc-7345), p-JNK (sc-7345), Beclin 1 (sc-48341), MAPLC3b (sc-271625), and SQSTM1/p62 (sc-48402) were used at 1:200 dilution (Santa Cruz Biotechnology, Dallas, TX, USA).

### 4.7. Biochemical Assays

#### 4.7.1. Alkaline Phosphatase Activity

The colorimetric acid phosphatase assay kit (Ab83367; Abcam; Cambridge, UK) was used to determine the enzymatic activity of acid phosphatase (AP) in 350 µg testicular tissue homogenates. AP activity was extrapolated from a standard sample curve (U/mL).

#### 4.7.2. ATP Levels

The fluorometric ATP assay kit (Ab83355; Abcam; Cambridge, UK) was used to quantify the total ATP in 350 µg testicular homogenates at Ex/Em = 535/587 nm. Tissue ATP levels were calculated from an ATP standard curve (nmol).

#### 4.7.3. Caspase 3 Activity

The colorimetric Caspase-3 assay kit measured the caspase 3 enzymatic activity in 350 µg of protein samples at 400 nm (Ab3940; Abcam; Cambridge, UK). The fold change amongst the animal groups was calculated using the sham group data as a reference.

### 4.8. Relative mRNA Expression through Quantitative PCR Analysis

The total mRNA was isolated from harvested testes using TRizol (15596026; Invitrogen, Waltham, MA, USA) and reverse-transcribed using the high-capacity cDNA reverse transcriptase kit (4368814; Applied Biosystems, Foster City, CA, USA). The relative quantitative PCR was performed using TaqMan gene expression assays and the QuantStudio™ 5 Real-time PCR system (A28573; Thermo Fisher Scientific, Waltham, MA, USA) by following the manufacturer’s instructions. The expression of the following genes was assessed: *Becn1* (Rn00586976_m1), *Map1lc3b* (Rn00575883_m1), and *Sqstm1* (p62) (Rn00709977_m1). The β-actin (*ACTB*, Rn00667869_m1) mRNA expression was used as an internal control. The relative mRNA expression was calculated through the 2^−ΔΔCt^ method.

### 4.9. Statistical Analysis

Raw data were statistically analyzed using the GraphPad Prism 8 software (GraphPad Software, San Diego, CA, USA). Variations across the means of the 5 animal groups were compared using the one-way analysis of variance (ANOVA). This was followed by the Holm–Sidak test to compare animal group pairs and to calculate their adjusted *p* values. Data are presented as the mean ± standard deviation (SD) and were considered significant if *p*-value < 0.05.

## Data Availability

Data are contained within this article.
